# Consideration of Photoactivity of TiO_2_ Pigments via the Photodegration of Methyl Orange under UV Irradiation

**DOI:** 10.3390/ma15176044

**Published:** 2022-09-01

**Authors:** Shuolin Zhou, Junzhuo Bai, Keying Huang, Xinlu Ye, Yingqing Peng, Min Lei

**Affiliations:** School of Elementary Education, Changsha Normal University, South Campus, No. 9, Xingshateli Road, Changsha 410100, China

**Keywords:** titanium dioxide pigment, methyl orange, photoactivity, photodegradation, mechanism

## Abstract

Developing a rapid and reliable method for measuring the photoreactivity of TiO_2_ pigments is of great importance for industrial application. The photoactivity of industrial TiO_2_ pigments were evaluated via the photodegradation of a model azo dye, methyl orange (MO), in the present work. The TiO_2_ pigments were characterized by Fourier-transform infrared spectroscopy (FTIR), ultraviolet–visible (UV–vis) spectroscopy, scanning electron microscopy (SEM), and photoluminescence (PL) spectroscopy. The photoactivity test results showed that the anatase TiO_2_ pigment was responsible for accelerating MO degradation, while the rutile pigment acted as a stabilizer, and effective UV absorber retarded the photodegradation of MO. It was found that the photodegradation of MO was driven mainly by photoholes (h^+^) and hydroxyl radicals (•OH), in the presence of TiO_2_ pigment with high photoactivity. With the help of the degradation intermediates during the photodegradation process and the calculated data, the preliminary degradation mechanism including azo bond cleaving, h^+^ oxidation, and hydroxylated products’ generation for MO was also elucidated. The photoactivity of TiO_2_ pigments can be rapidly evaluated in this work, which would be an efficient approach for assessing the product quality control and the end-use performance of TiO_2_ pigments.

## 1. Introduction

Titanium dioxide (TiO_2_) is an important chemical material that is extensively utilized in coatings, rubber, plastics, and paper due to its excellent properties of higher whiteness, gloss, and refractive index, which better cover power and UV absorption performance [1,2]. The global production of TiO_2_ reached almost 8.0 million tons in 2021 [3], and it is projected to reach 8.8 million tons by 2025 [4]. The biggest consumer of TiO_2_ is the pigment industry, which is ca. 95% of the world’s consumption of titanium dioxide [5].

It is well-known that TiO_2_ can generate electron-hole pairs when irradiated with photons, with energy that is equal to or greater than its band gap energy [6,7]. The generated reactive species, such as holes (h^+^), hydroxyl radicals (•OH), and superoxide radicals (O_2_^•−^), are commonly believed to be highly reactive radicals for the decomposition of organic molecules [8,9,10,11]. If bare TiO_2_ is directly used in outdoor application, the photocatalyzed behavior of TiO_2_ will lead, either directly or indirectly, to the organic materials’ discoloration or even total destruction [12,13]. The applications of pure TiO_2_ particles with a high level of photocatalytic activity were severely limited in the paint, coating, and plastic [14]. In order to suppress the photoactivity of titanium dioxide, while maintaining its UV-shielding performance, inert oxides such as SiO_2_ [15,16], Al_2_O_3_ [17,18], ZrO_2_ [19], and CeO_2_ [20] and organic treatments have usually been applied to coat the titanium dioxide surface. Several recent studies, however, have demonstrated that the photoreactivity of titanium dioxide pigment remains one of the most important influential factors for coatings degradation [13,21,22].

Previous studies have focused on various physical changes such as weight loss, discoloration, and gloss retention in a coating film, through an artificial accelerated aging test or outdoor exposure test to evaluate the photoactivity performance of industrial TiO_2_ pigments [23,24,25]. These physical changes, however, often take hundreds or thousands of hours to appear. Besides, the analysis of intermediate products about such a solid sample is difficult. In recent years, the formation of CO_2_ (ca. 2360 cm^−1^) that is caused by the photocatalytic oxidation in a polymer film can be monitored by FTIR spectroscopy [26]. Other methods, such as time-resolved photoluminescence spectroscopy measurement [27], electron paramagnetic resonance (EPR) analysis [28], and the photoelectrochemical approach [29] have also been proposed. However, the above-mentioned studies required tedious operation, a long amount of time, or expensive equipment. In addition, the test method, which used Rhodamine-B or Acid Blue 9 degradation to evaluate the photostability of TiO_2_ particles, uncoated or coated by metal oxides, also required a very long measurement time [30,31]. It is worth noting that some characteristic results of the photoactivities of various commercial TiO_2_ pigments are usually ambiguous [32]. Thus, it seems pretty difficult to assess and select the appropriate TiO_2_ pigments because little information about the photoactivity of TiO_2_ pigments was provided from the perspective of the degradation mechanism [33]. Therefore, developing a rapid and reliable method for measuring the photoreactivity of TiO_2_ pigment would be desirable.

In the present work, commercial TiO_2_ pigments were used and characterized by ultraviolet-visible (UV–vis), Fourier-transform infrared (FTIR), scanning electron microscopy (SEM), and photoluminescence (PL) spectroscopy. The apparent photoactivities of TiO_2_ pigments were evaluated utilizing methyl orange (MO), which acted as a model molecule under ultraviolet irradiation in an aqueous solution. During the photodegradation process, the contributions of reactive species, e.g., holes and hydroxyl radicals, were further investigated. The preliminary degradation mechanism of MO was also elucidated via the identified intermediates by gas chromatography–mass spectrometry (GC–MS) and theoretical calculation by Gaussian. This is expected to identify the various TiO_2_ pigments and provide the referential information for the application of TiO_2_ pigments.

## 2. Experiments

### 2.1. Materials

The industrial TiO_2_ pigments were purchased from Hunan Zhuzhou Chemical Group Co., Ltd. (Zhuzhou, China) and labeled as P1, P2, and P3 in this work, with their corresponding information listed in Table 1. Methyl orange (C_14_H_14_N_3_SO_3_Na, purity > 99%) was obtained from Tianjin Damao Chemical Reagent Co. Inc. Other chemicals such as silver nitrate (AgNO_3_), potassium iodide (KI), methanol (MeOH), and isopropanol (IPA) were of analytical grade and were used as received without further purification. Water was prepared by a Millipore Milli-Q ultrapure water-purification system.

### 2.2. TiO_2_ Pigment Characterization

The FTIR spectra of the samples were collected by the KBr pellet technique on a Nicolet 370 Fourier-transform infrared spectrophotometer in the range of 500–4000 cm^−1^. The morphologies of the samples were tested by scanning electron microscopy on a MIRA3 TESCAN using an accelerating voltage of 20 kV. The UV–vis transmittance spectra of TiO_2_ pigments were obtained on a Hitachi U-3010 spectrophotometer with an integrating sphere. PL spectra were obtained on a Hitachi F-4500 fluorometer with the excitation at 300 nm, slit width of 5.0 nm, multiplier voltage of 700 V, and scanning region of 300–550 nm.

### 2.3. Photoactivity Tests

**Figure 1:**
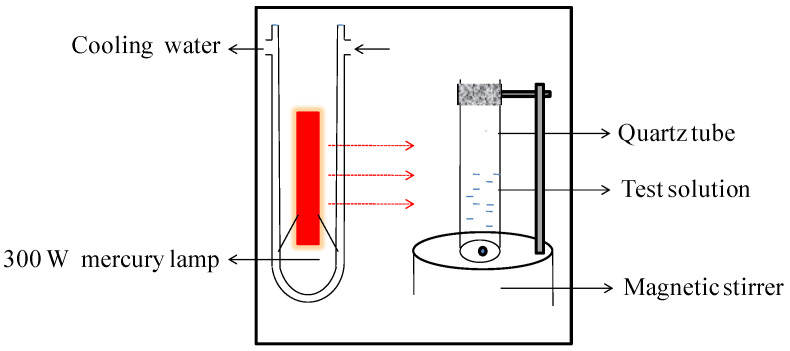
The apparatus for photoactivity performance testing.

The photoactivity tests were carried out in a quartz reactor (total volume ca. 70 mL). The light source was a 300 W medium-pressure mercury lamp (λ*_max_* = 365 nm, Philips, Saarbrücken, Germany) with a double-walled cooling-water jacket housed in one side of the reactor to provide the irradiation. The experimental device is schematically shown in Figure 1.

A volume of 35 mL of solution containing 30 μmol/L of MO was added to the quartz reactor with 35 mg of TiO_2_ pigment powder. Before exposure to UV radiation, the suspension was magnetically stirred in the dark for 30 min. During the photodegradation process, about 2 mL of the reaction solutions was obtained at regular intervals and centrifuged to remove TiO_2_ pigment. The concentration of MO was analyzed using UV-vis spectrophotometer and calculated from the standard calibration for the characteristic λ*_max_* of MO. The degradation efficiency was calculated according to the equation: X (%) = (*C*_0_ − *C*_t_)/*C*_0_ × 100%, where *C*_0_ is the initial concentration of MO, and *C*_t_ is the concentration of MO after photoirradiation for t minutes. All the MO degradation experiments were performed at least in triplicate, and average data are presented.

The degradation intermediates in the photodegradation process were identified by gas chromatography–mass spectrometry (GC–MS) method. Shimadzu GC system equipped with a capillary (30 m length, 0.32 mm internal diameter, 0.25 μm film thickness) was used. Helium was used as a carrier gas at a flow rate of 1 mL/min. The oven temperature program was as follows: 3 min isothermal at 100 °C, ramped from 100 to 260 °C at a rate of 10 °C/min, and held at 300 °C for 10 min. The NIST05S.LIB library was used for the mass spectrum analysis.

### 2.4. Theoretical Calculations

Theoretical calculations were carried out by density functional theory (DFT) method (B3LYP) with the 6-31G (d,p) basis set using the Gaussian 09 program (Gaussian, Inc., Wallingford, CT, USA). The point charges (PC) of MO were calculated to predict the chemisorption position of MO onto the surface of TiO_2_ pigment.

## 3. Results and Discussions

### 3.1. TiO_2_ Pigment Characterization

**Figure 2:**
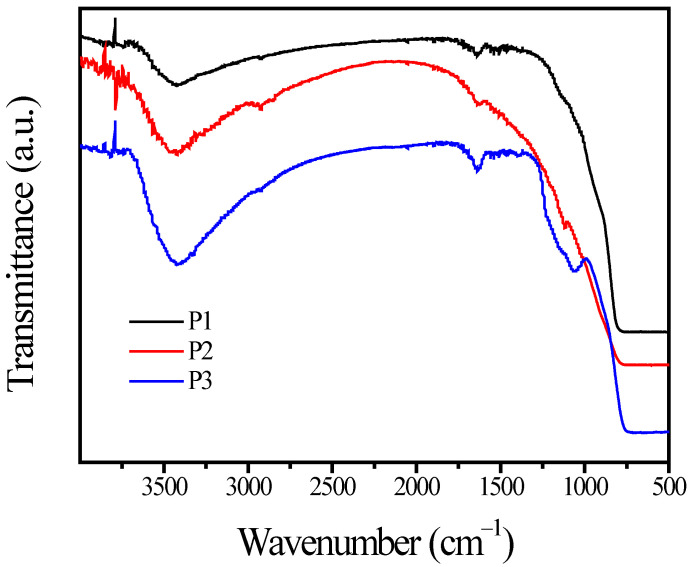
FTIR spectra of the samples.

The FTIR spectra of TiO_2_ samples are displayed in Figure 2. The broader peak at 3460 cm^−1^ was attributed to the stretching vibration and bending vibration of hydroxyl groups [34]. The other peak at 1630 cm^−1^ was attributed to the bending vibration of H-O-H. In addition, a peak in the range of 500–800 cm^−1^ is generally related to the stretching vibration modes of Ti-O-Ti bands [35]. In the FTIR spectra, both P1 and P3 showed new peaks at 1080 cm^−1^ and 1600 cm^−1^, which are characteristic of SiO_2_ and Al_2_O_3_ [36]. However, the photoholes (h^+^) induced by UV light would oxidize surface hydroxyl groups onto the TiO_2_ surface to generate •OH radicals, if the TiO_2_ surface was not coated completely. In that case, the TiO_2_ pigments are likely to participate in organic substrate degradation under UV irradiation.

**Figure 3:**
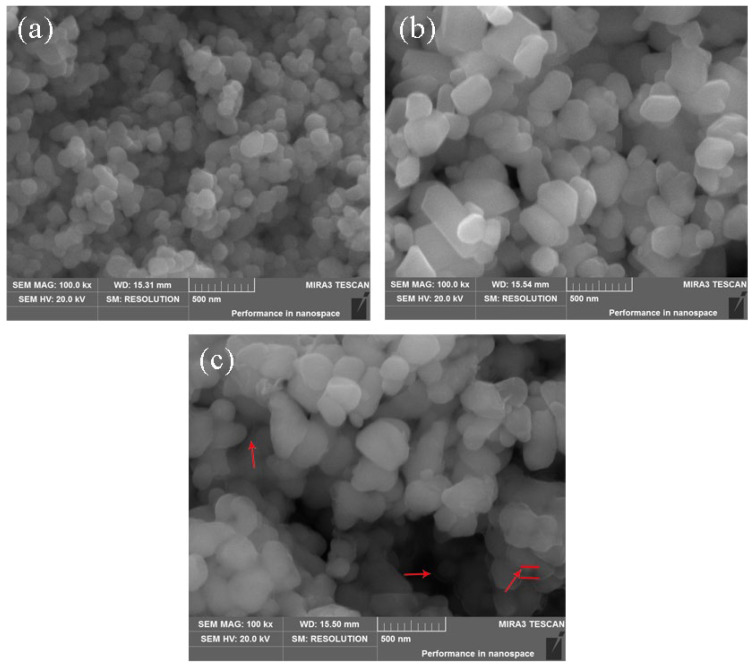
SEM images of the samples: (**a**) P1; (**b**) P2; (**c**) P3.

SEM images of TiO_2_ pigments are shown in Figure 3. The shape of P1 was spherical, and the size of P1 was approximately 200 nm (Figure 3a). Figure 3b clearly demonstrates that the size range of P2 was widely from several micrometers to tens of micrometers, and P2 has a relatively smooth surface. As indicated by the arrow in Figure 3c, the surface of TiO_2_ was coated by amorphous silica and alumina coating layers, which was consistent with the FTIR analysis.

**Figure 4:**
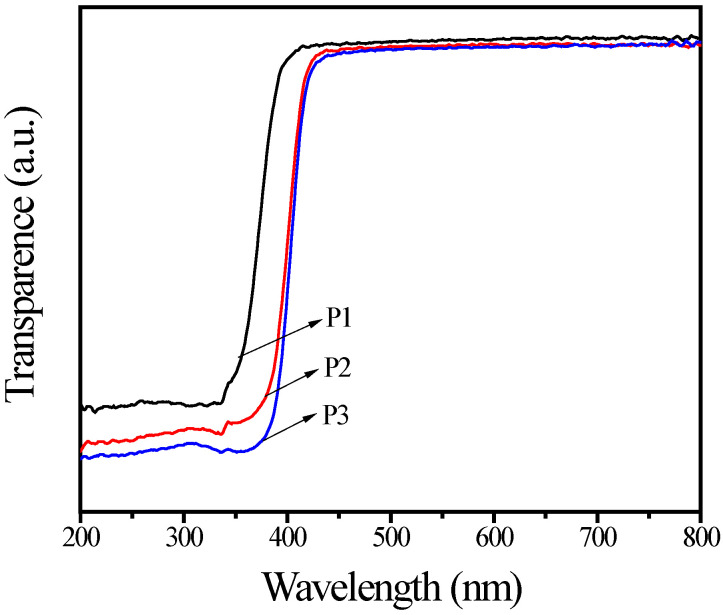
UV–vis spectra of the samples.

UV–vis spectra of TiO_2_ pigments are shown in Figure 4. The increase in transmittance in the UV region (280–400 nm) suggests the decrease in UV-shielding ability. The UV-shielding ability of P3 decreased slightly compared to the original P2. This may be because the point defects in both amorphous Al_2_O_3_ and SiO_2_ on the surface of the TiO_2_ particles can absorbe UV light in this region [37,38]. The UV-shielding ability of the rutile TiO_2_ pigment P3 was better than that of the anatase TiO_2_ pigment P1, because the former could absorb UV light up to 400 nm, while the latter only showed significant absorption up to 380 nm. Importantly, the rutile phase was likely to be much lower apparent photoreactivity than the anatase phase under UV irradiation [13]. Thus, the rutile-based pigment with UV absorption performance and lower photoreactivity can act as an effective UV absorber in the polymer coatings.

**Figure 5:**
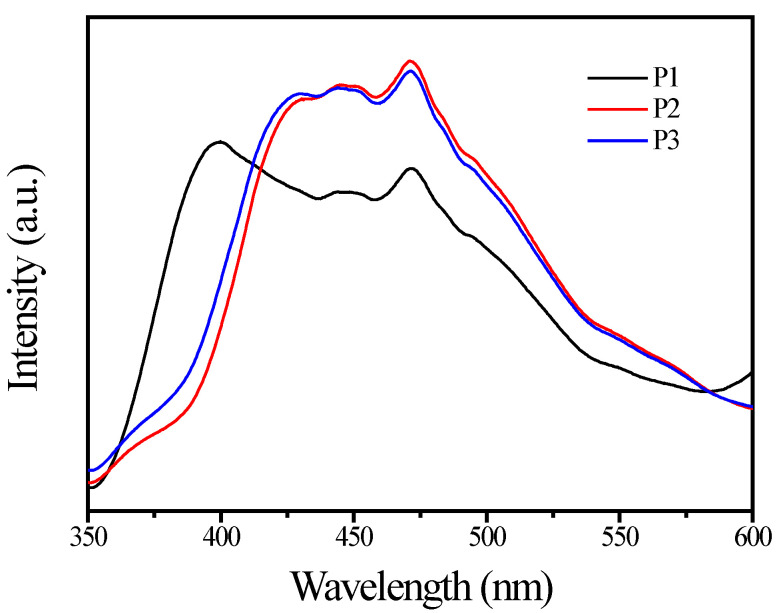
PL spectra of the samples with the excitation wavelength of 300 nm.

Figure 5 displays the PL spectra of TiO_2_ pigments. It is known that the PL signals of semiconductor particles mainly result from the recombination of photoinduced charge carriers. Commonly, lower PL intensity indicates a lower recombination rate of photoinduced electron–hole pairs and, thus, higher photocatalytic activity [39,40,41]. The anatase-based pigment P1 gave a strong emission band with a wavelength maximum at 390 nm, whereas the rutile-based pigment for both P2 and P3 gave strong emission bands with a wavelength at ca. 415 and ca. 470 nm, respectively. These differences may be attributed to the different crystalline structure of TiO_2_ particles. It can be seen that the SiO_2_/Al_2_O_3_ coating layer did not influence the PL spectra of TiO_2_ pigment. Several excitonic PL peaks illustrated that the surface states or defects of the TiO_2_ pigment were abundant [27].

### 3.2. Photoactivity Tests

**Figure 6:**
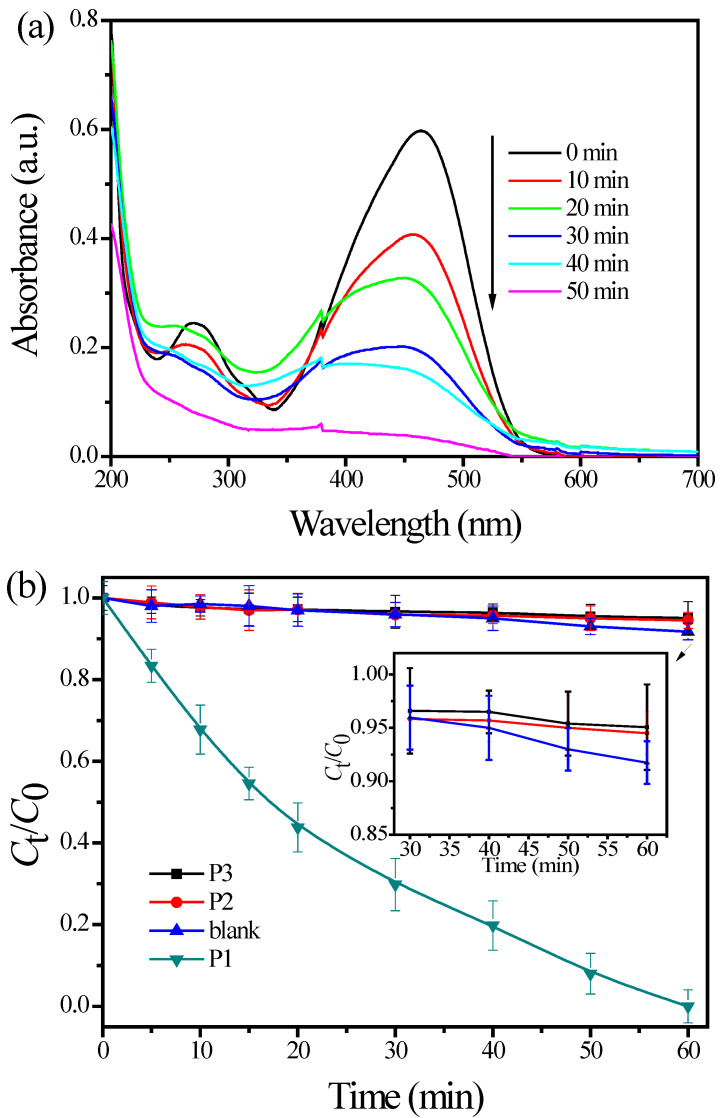
(**a**) Typical UV–vis spectra of MO during the photocatalytic degradation process at the initial concentration 30 μmol/L in an aqueous pigment P1 (1.0 g/L) and natural pH; (**b**) photodegradation curves of MO over different TiO_2_ pigments under UV light irradiation.

The maximum absorption at 464 nm is a characteristic peak of the π-π* transition of azo bonds (-N=N-). The peak at 280 nm corresponds to the π-π* transition of the benzyl ring. As shown in Figure 6a, the peak at 464 nm shifted towards the low absorbance values with the increase in illumination time for P1. As described above, the loss in the spectral features of the MO reveals the start of decomposition. However, a blank experiment was performed under UV irradiation in the absence of TiO_2_ pigments, and the degradation rate was 7.0% in 50 min. The photodegradation efficiency of MO over various TiO_2_ pigments was compared, and the results re shown in Figure 6b. Both P2 and P3 exhibit the negligible activity for photodegradation of MO. Moreover, P3 showed a more significant inhibitory effect for the degradation of MO than that of P2, suggesting that the coated rutile pigments with suppression of photoactivity can act as a stabilizer and effective UV absorber [42]. On the contrary, the TiO_2_ pigment P1, the form of anatase, has a significant photoreactivity under UV irradiation, in spite of its surface being coated by SiO_2_/Al_2_O_3_, showing 95.0% photocatalytic degradation for MO after 50 min of irradiation. The above-mentioned results suggest that the photoactivity of the commercial TiO_2_ pigments can be rapidly evaluated and decrease in the order P1 > P2 > P3. These experimental results were consistent with our previously reported findings [43].

### 3.3. Photodegradation Mechanism

**Figure 7:**
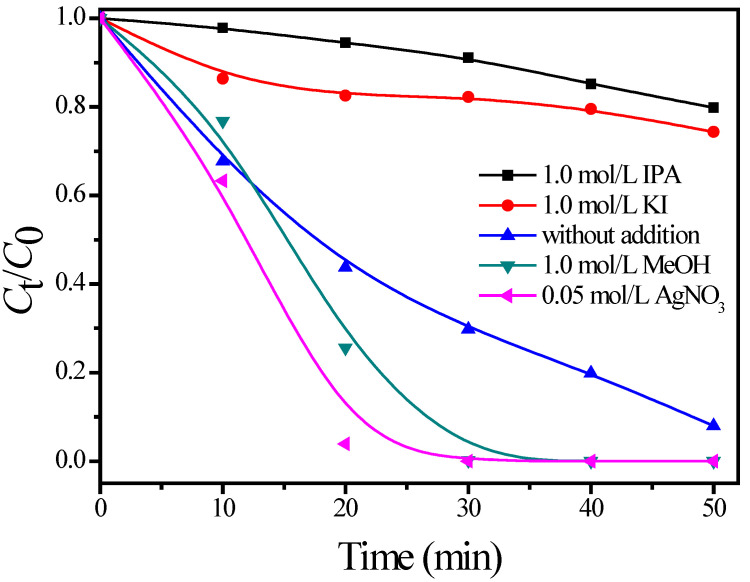
Effects of various reactive species scavengers on the photodegradation of MO by pigment P1.

The scavengers for holes and hydroxyl radical were further employed to investigate the photoactivity of TiO_2_ pigments. When KI, which was often used as a hole-capturer [44], was added to the reaction system, the degradation efficiency of MO was suppressed and reduced to 74.4%. As a well-known hydroxyl radical scavenger, the addition of IPA decreased the degradation efficiency to 79.8%. MeOH was reported to serve as an alternative hole and hydroxyl radical scavenger [45]. The degradation rate of MO, however, was significantly improved when 1.0 mol/L methanol was added, which seems to be in contradiction to the expected result. This observation can be explained on the basis that a large excess of methanol reacts rapidly with both h^+^ and •OH, generating •OCH_3_ radicals that can be trapped by O_2_. As a result, HO_2_• radicals were generated, which are of an important intermediate active species in the solution. The photodegradation of MO was further investigated in the presence of an electron scavenger (Ag^+^ ions). Figure 7 depicts that the addition of Ag^+^ ions can greatly promote the degradation of MO. In total, 96.1% of MO was degraded after irradiation for only 20 min. This is because the silver ion is a strong electron acceptor [*E*^0^(Ag^+^/Ag) = 0.80 V_NHE_] that increases the lifetime of holes and electrons [46], resulting in improving the oxidation rate of MO. The above-mentioned results indicate that the degradation of MO was driven by h^+^ and •OH radicals.

**Figure 8:**
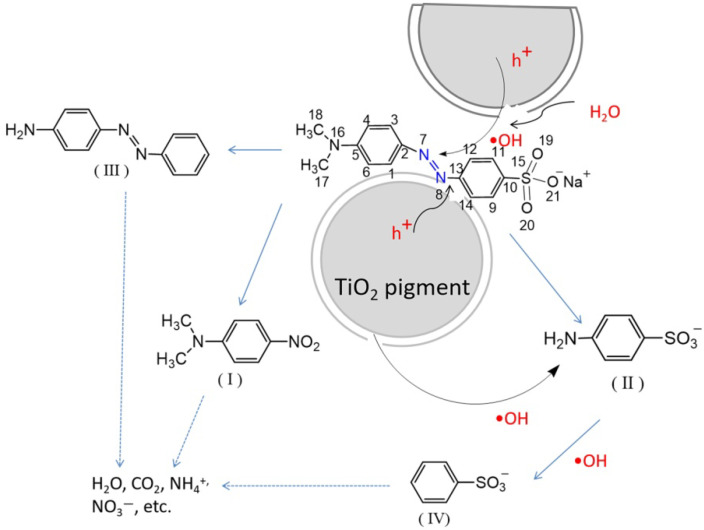
Proposed pathways of photodegradation of MO.

After the exploration of active species during the MO photodegradation process, we also tried to elucidate the photodegradation mechanism of MO in the presence of a highly photoactive pigment. The experiments were conducted using 30 μmol/L solutions of MO in the presence of P1 under UV irradiation, and the main degradation intermediates were identified by GC–MS. The GC–MS chromatogram is given in Figure S1. The *m/z* peak at 304 can be attributed to the ionization of the dye molecule. Two degradation intermediates with *m/z* peaks at 166 (denoted as compound I) and 172 (denoted as compound II) may be attributed to the substituted aromatic amines. The theoretical atomic point charges of MO were computed to predict initial attack site, and the corresponding data are listed in Table 2. It can be seen from the table that the most negative point charges were located at C2 (−0.886), C13 (−0.776), while a more positive charges was found for N7. The azo group (-N=N-), therefore, was apt to be absorbed on the surface of TiO_2_ pigments, attacked by photoholes, and decomposed. Additionally, the electrophilic nature of •OH radicals was also apt to attack the azo group, which has the higher electron density [47]. So, the characteristic peak at 464 nm (in Figure 6a), shifting toward the low absorbance values, increased in the duration time of illumination. The cleavage of the -N=N- double bond was consistent with the previous results [45,48]. Besides, MO can be adsorbed on the TiO_2_ pigment’s surface via the sulfonate group, due to the negative point charges located on the oxygen atoms of O19, O20, and O21 [49]. The photodegradation pathways for MO are shown in Figure 8. •OH radicals (oxidation potential: 2.8 V) are stronger oxidants that can oxidize organics by the abstraction of protons generating organic radicals (R•), which can be further oxidized [48]. Moreover, the S15–C10 bond (1.8431 Å), N16–C17 bond (1.4735 Å), and N16–C18 bond (1.4737 Å) are longer than the other C–C bonds (ca. 1.44 Å), which indicated that the corresponding groups were more easily removed than the C–C groups. The degradation intermediate with *m/z* = 197 (denoted as compound III) may be attributed to the loss of the sulfonic group and methyl group during the photodegradation process. The degradation intermediate with *m/z* = 156 (denoted as compound IV) may be generated from (II) by further loss of -NH_2_. Eventually, the mineralization of MO by stepwise advance oxidation leads to the production of small molecules and ions such as CO_2_, H_2_O, SO_4_^2−^, NO_3_^−^, NH_4_^+^, etc. [50].

These results indicated that •OH radicals and photoholes exhibited the major contributions to the degradation of MO, which provides powerful evidence for the photocatalytic performance of TiO_2_ pigment. This is mainly due to the fact that the SiO_2_/Al_2_O_3_ coating layers did not completely cover all the TiO_2_ surface in the industrial production process. The roles of TiO_2_ pigments relating to the paint film would also be predicted in actual usage using this method. It is, therefore, believed that a TiO_2_ pigment with high photoactivity could accelerate the degradation of organic substrates in polymer film [51], which will possess a series of unfavorable properties, such as surface gloss loss, color change, chalking, and cracking.

## 4. Conclusions

In summary, the photoactivity of commercial TiO_2_ pigments was quickly evaluated via the photodegradation of MO. The photodegradation results showed that rutile TiO_2_ pigment, along with high UV opacity and photostability, retards the photodegradation of MO, whereas the anatase TiO_2_ pigment operates primarily as an effective photoactivity for accelerating the rate of photodegradation. h^+^ and ·OH radicals are the major reactive species in the photodegradation of MO over anatase TiO_2_ pigment. We also reveal the photodegradation mechanism for MO, which is supported by GC–MS and theoretical calculation. This work provides a useful strategy toward evaluating the photoactivities of various TiO_2_ pigments and assessing the potential effects of TiO_2_ pigments on coatings stabilization or degradation. At the same time, this method may be very useful in preparing multi-purpose TiO_2_ pigments.

## Figures and Tables

**Table 1:** Main characterization of industrial TiO_2_ pigments.

TiO_2_ Pigment Sample	Composition	Surface Treatment	Applications
P1	TiO_2_ (anatase, >98%)	SiO_2_/Al_2_O_3_	Paints, fiber, rubber, metallurgy, etc.
P2	TiO_2_ (rutile, >98%)	None	-
P3	TiO_2_ (rutile, >92%)	SiO_2_/Al_2_O_3_	Coatings, rubber, plastic, etc.

**Table 2:** Point charges and bond length on atoms of MO at the B3LYP/6-31+G(d,p) level.

Atom Label	Point Charges	Bond	Bond Length (Å)
C1	0.239	C1–C2	1.4419
C2	−0.886	C2–C3	1.4369
C3	0.070	C3–C4	1.3639
C4	−0.070	C4–C5	1.4419
C5	0.402	C5–C6	1.4471
C6	−0.138	C6–C1	1.3618
N7	0.055	N7–C2	1.3356
N8	−0.088	N7–N8	1.3267
C9	−0.214	C9–C10	1.4593
C10	−0.637	C10–C11	1.4613
C11	0.066	O11–C12	1.3543
C12	0.550	O12–C13	1.4505
C13	−0.776	C13–C14	1.4455
C14	0.060	C14–C9	1.3552
S15	1.341	S15–C10	1.8431
N16	−0.079	N16–C5	1.3401
C17	−0.285	N16–C17	1.4735
C18	−0.276	N16–C18	1.4737
O19	−0.467	S15–O19	1.4557
O20	−0.465	S15–O20	1.4558
O21	−0.320	S15–O21	1.6345

## Data Availability

All data generated or analyzed during this study are included in this manuscript.

## Supplementary Materials

The following supporting information can be downloaded at: https://www.mdpi.com/article/10.3390/ma15176044/s1, Figure S1: The total ion chromatogram of the degradation products.
