# Supplementary material for: Consideration of Photoactivity of TiO2 Pigments via the Photodegration of Methyl Orange under UV Irradiation
Source: Materials (Basel). 2022 Sep 1;15(17):6044. doi: 10.3390/ma15176044 (PMC9457480; doi:10.3390/ma15176044)
Supplement: Supplementary file 1 [file materials-15-06044-s001.zip › materials-1868271-supplementary.pdf]

Supplementary Material

Consideration of Photoactivity of TiO_2 pigments via the Photodegration of Methyl Orange under UV Irradiation

Shuolin Zhou *, Junzhuo Bai, Keying Huang, Xinlu Ye, Yingqing Peng and Min Lei

School of Elementary Education, Changsha Normal University, South Campus, No. 9, Xingshateli Road, Changsha 410100, China
* Correspondence: slzhou1989@163.com

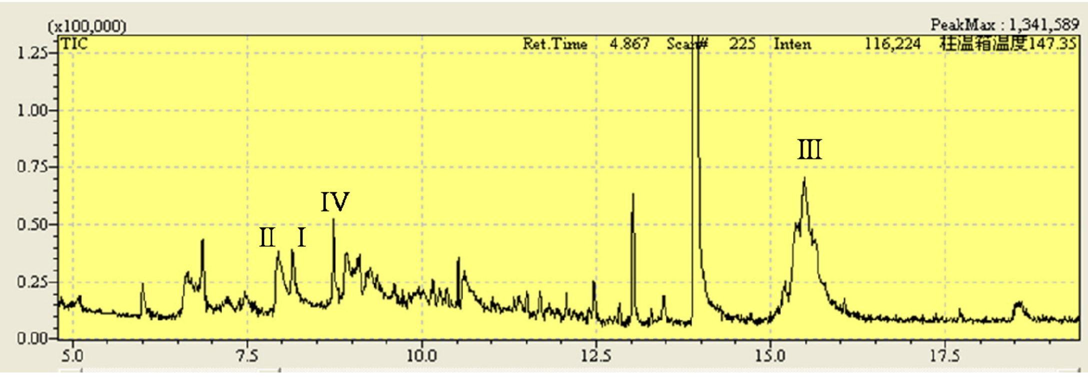

Figure S1. The total ion chromatogram of the degradation products. The degradation intermediates with m/z =166 denoted as compound I, m/z = 172 denoted as compound II, m/z = 197 denoted as compound III and m/z = 156 denoted as compound IV.
